# Supplementary material for: Predicting visual function by interpreting a neuronal wiring diagram
Source: Nature. 2024 Oct 2;634(8032):113–23. doi: 10.1038/s41586-024-07953-5 (PMC11446822; doi:10.1038/s41586-024-07953-5)
Supplement: Supplementary file 6 — CRF and ERF predictions for individual TmY4 and TmY9 cells. Analogous to Supplementary Data 3, but for TmY target types. Shown are the top four monosynaptic pathways, the strongest pathway passing through each of the top ten intermediary types (ranking from Extended Data Fig. 7), and the trisynaptic pathway Tm1–TmY–Dm3–TmY (see the section entitled Prediction of spatial normalization). [file 41586_2024_7953_MOESM6_ESM.zip › DataS4/TmY9qΓèÑ/720575940621700528.pdf]

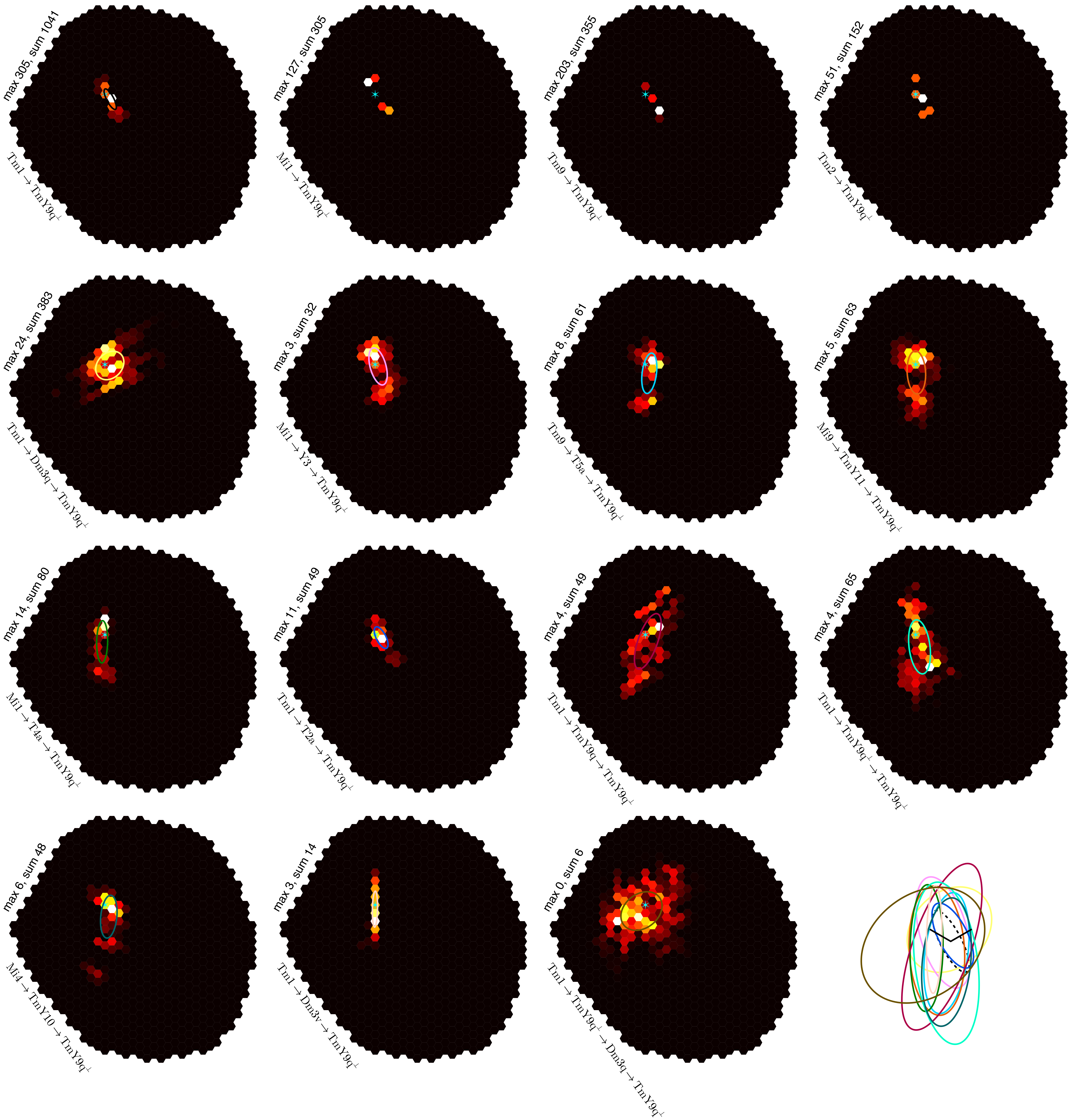

max 305, sum 1041
Tm1 → TmY9q⊥
max 127, sum 305
Mi1 → TmY9q⊥
max 203, sum 355
Tm9 → TmY9q⊥
max 51, sum 152
Tm2 → TmY9q⊥
max 24, sum 383
Tm1 → Dm3q → TmY9q⊥
max 3, sum 32
Mi1 → Y3 → TmY9q⊥
max 8, sum 61
Tm9 → T5a → TmY9q⊥
max 5, sum 63
Mi9 → TmY11 → TmY9q⊥
max 14, sum 80
Mi1 → T4a → TmY9q⊥
max 11, sum 49
Tm1 → T2a → TmY9q⊥
max 4, sum 49
Tm1 → TmY9q⊥ → TmY9q⊥
max 4, sum 65
Tm1 → TmY9q⊥ → TmY9q⊥
max 6, sum 48
Mi4 → TmY10 → TmY9q⊥
max 3, sum 14
Tm1 → Dm3v → TmY9q⊥
max 0, sum 6
Tm1 → TmY9q⊥ → Dm3q → TmY9q⊥
